# Alkaloid profile of Italian alpine milk

**DOI:** 10.1111/1750-3841.70027

**Published:** 2025-02-04

**Authors:** Tiziana Nardin, Edi Piasentier, Alberto Romanzin, Francesca Martinelli, Roberto Larcher

**Affiliations:** ^1^ Centro Trasferimento Tecnologico, Fondazione E. Mach San Michele all'Adige Italia; ^2^ Dipartimento di Scienze Agrarie ed Ambientali (DISA) Università di Udine Udine Italia

**Keywords:** alkaloids, alpine, milk

## Abstract

**Abstract:**

This study examines the alkaloid profiles in alpine milk. Alkaloids could pose a health concern but also prove interesting from a geographic traceability perspective. Over three consecutive days, 48 daily milk samples were collected from 16 lactating cows grazing on two alpine pastures in Northeast Italy, with 8 cows from each pasture. Simultaneously, alpine herbs selecting by the cows during grazing were collected using the hand‐plucking technique. Additionally, 12 milk mass samples were obtained from an entire herd of 110 cows. Liquid chromatography coupled with high‐resolution mass spectrometry was employed to analyze both herbage and milk samples, identifying 41 alkaloids with pure standards and putatively identifying another 116. The results revealed a transfer of 0.4% for pyrrolizidine alkaloids, 2.7% for indole alkaloids, and 12% for steroidal alkaloids from herbs to milk. A partial least squares—discriminant analysis model based on the alkaloid profiles achieved a correct reclassification of 67% of milk samples from cows grazing on the two distinct pastures. Despite the minimal transfer, which should be considered positive in terms of health, it opens the door to interesting studies on the use of alkaloids as traceability markers for mountain products.

**Practical Application:**

The study provides a novel perspective interaction between alpine grazing systems and milk composition. This research could be useful for enhancing mountain pasture products in terms of healthiness and can help prevent fraud on the declaration of origin.

## 1 INTRODUCTION

Alkaloids (alks), a diverse group of naturally occurring chemical compounds with potent pharmacological properties, have garnered significant attention in recent years due to their presence in various food products, including dairy products. The transfer of alks into dairy products raises important questions regarding food safety and potential health implications for consumers. In fact,  investigation of alk focus on particular toxic alks, such as pyrrolizidines (Pyzs), tropanes (Trps), or quinolizidines (Qnzs) (Engel et al., 2022; Klein et al., 2022; Lamp et al., 2021), whose presence in milk is mainly derived from contaminated animal feed products. For example, the transfer of pyrrolizidine alks from contaminated feed to milk in cows has been established (Hoogenboom et al., 2011), and jacoline, a minor component in ragwort, emerged as a major pyrrolizidine alk in milk, with an estimated carry‐over of 4% compared to an overall 0.1% transfer rate for the alks.

The toxicity of alks mainly depends on their chemical structure and how they interact with the body. Many alks have strong effects on the central nervous system by interfering with receptors and cellular functions. For example, alks such as scopolamine and atropine (which belong to the tropane class) act on muscarinic receptors in the brain and peripheral nervous system, causing effects such as pupil dilation, tachycardia, and altered motor function. Other alks, such as those in the pyrrolizidine alks (Pyzs) family, can be hepatotoxic. When ingested, these alks are metabolized in the body to form toxic metabolites that damage liver cells, potentially leading to cirrhosis, liver failure, or, in extreme cases, death (Kohnen‐Johannsen and Kayser, 2019; Wang et al., 2021). Now, there are no specific limits for alks in milk, and common regulatory authorities such as the European Food Safety Authority (EFSA), the U.S. Food and Drug Administration (FDA), and Codex Alimentarius may have specific guidelines and limits for certain specific toxic alks. In particular, an acute reference dose (ARfD) for the sum of l‐hyoscyamine and scopolamine was established at a level of 0.016 µg kg^−1^ body weight (Perharič et al., 2013).

In contrast, not all alks are harmful, some, such as morphine and quinine, have beneficial effects and are used in medicine for pain relief and the treatment of diseases like malaria (Duarte et al., 2010; Dugan et al., 2024). Herbs may contain different chemical classes of alks, often characteristics for different plant families (Nardin et al., 2023). Considering the natural consumption of pasture herbs by livestock, multiple classes of alks could naturally be found in animal‐derived products like milk. Now, there is no extensive information in the literature on the alk profile in dairy products. The presence of alks in milk is important not only from a safety perspective but also in terms of the traceability of milk origin. The chemical profiles of alks in milk can offer insights into the type of pasture consumed by cows, distinguishing milk from highland areas where certain herbs may be more prevalent, from milk produced in lowland pastures. This differentiation could have implications for both consumer health and market authenticity (Jin et al., 2023).

Milk, in fact, is certainly one of the most important foods. In 2023, the world produced 950 million tons of raw milk, with Europe contributing 234 million tons, of which 227 million tons were cow's milk, and Italy produced about 13 million tons. (Dairy market review—Emerging trends and outlook in 2023; https://efaidnbmnnnibpcajpcglclefindmkaj/ https://openknowledge.fao.org/server/api/core/bitstreams/68f7f25d‐b3cb‐418e‐b04d‐5708e5bcea1e/content). As mandated by Regulation (EU) No. 1169/2011, Italy, through the interministerial decree of December 9, 2016, made the indication of the origin of raw milk material mandatory on the packaging label. Similarly, not only is the country of origin important, but also the differentiation of milk from either highland or lowland areas is relevant to the quality aspect. The control of fraud in origin declarations is crucial for ensuring transparency and consumer trust in food products, especially in the dairy industry. Accurate labeling of the origin of milk helps protect both consumers and producers from deceptive practices, such as misrepresenting the source of the product. Fraudulent claims about the origin can undermine local industries, disrupt fair trade, and lead to economic losses for legitimate producers. Furthermore, the correct declaration of origin is vital for maintaining food safety standards, as certain regions may have specific regulations and certifications that ensure higher quality and traceability. Stringent controls and verification systems are necessary to combat these frauds and maintain the integrity of the food supply chain.

In this context, the objective of this study was to investigate the transfer of alks from alpine pastures to milk, with a particular focus on identifying the alk profiles in milk samples from 16 cows grazing in two distinct alpine regions. This study aims to assess how different grazing environments influence the alk content in milk, to explore the potential toxicity risks, and to examine the use of these alk profiles as markers for geographical origin and product authenticity.

## 2 MATERIALS AND METHODS

### 2.1 Reagents and solutions

LC‐MS grade acetonitrile (ACN), LC‐MS grade methanol (MeOH), MS grade formic acid (FA, 98%), HPLC grade hexane 97%, and LC‐MS grade ammonium acetate were purchased from Fluka, and ammonia solution 25% was purchased from Merk Millipore. For mass calibration, a standard mix of n‐butylamine, caffeine, methionine‐arginine‐phenylalanine‐alanine (MRFA), and Ultramark 1621 (Pierce ESI Positive Ion Calibration Solution) was used. Deionised water (H_2_O) was produced with an Arium Pro Lab Water System (Sartorius AG). Alk standards aconitine, atropine, coniine, echimidine, erucifoline, erucifoline‐N‐oxide, gramine, heliotrine, hyoscyamine, jacobine, jacobine‐N‐oxide, jervine, monocrotaline, lasiocarpine, lycopsamine, protoveratrine A, retrorsine, retrorsine N‐oxide, scopolamine, seneciphylline, senecionine, senecionine N‐oxide, senecivernine, senkirkin, sipeimine, alpha‐solamargine, solasodine, alpha‐solasonine tomatidine, tomatine, veratramine, and veratridine were purchased from PhytoLab GmbH & Co. KG, while caffeine, harmaline, nicotine, quinine, quinidine, strychnine, theobromine, and theophylline were purchased from Sigma.

A mix solution (1 mg L^−1^ each single alk) was set out starting from individual stock solutions of each alk (100 mg L^−1^) prepared by dissolving the standard powder in an aqueous methanol solution (50:50, by vol) and used for calibration in the range 0.02–500 µg L^−1^, injecting 1 µL for each level. The mix solution was prepared freshly before each analysis, while stock solutions were stored at −4°C.

### 2.2 Flora characterization

The study was carried out on a traditional alpine farm in north‐eastern Italy (Malga Montasio; 46°24′45″N, 13°25′53″E) during the summer grazing period. Two different types of pasture were considered, *Poion alpinae* (PO), a nutrient‐rich pasture located at 1500  m above sea level (a.s.l.), and *Seslerion caeruleae* (SE), a nutrient‐poor one at 1700  m a.s.l. (Bovolenta et al., 2014).

Characterization of the flora was performed by an experienced botanist in 100 m^2^ sample areas at the beginning of the grazing period, according to the different altitude of pastures, with five and nine replicates on PO and SE, respectively, as described by Pasut (2016). Their replication frequency and average coverage in the two pastures are shown in Table S1.

### 2.3 Herbage and milk samples selection

A group of 16 lactating cows was selected from a herd of 110 Italian Simmental dairy cows grazed in two different pastures (eight for each pasture). Cows had access to pasture during the day and the night, and a grass intake is estimated at 16–18  kg dry matter (DM) day^−1^ (Gianelle et al., 2017). Moreover, a feed supplement for lactating cows was administered at milking time (twice a day), receiving in total 2.5 kg head per day of a mixed concentrate based on corn flour, barley flour, dried beet pulp, soybean meal, wheat bran, sugar beet molasses, sodium chloride, calcium phosphate, and sodium bicarbonate. Chemical composition was follows: crude protein 16%, ether extract 3.2%, crude fiber 10.5%, and ash 8.5%.

All the cows involved in the trial had between two and six lactations (mean 4.1), corresponding to an age of ∼6 years; were in good health; and had already grazed at this alpine farm for at least one season. The herbage selected by each cow was manually sampled, using a hand‐plucking technique that mimicked animal intake (Berry et al., 2002). The sampling was repeated for 3e consecutive days for both pastures, gathering 48 herbage mixtures. The number of replicates of the surveys is defined based on the surface and heterogeneity of the pasture. The weight of the grass samples collected with the hand plucking technique was variable (availability of grass, animal behavior, etc.), on average around 200–300 g. The same number equal to 48 milk samples were collected from the 16 cows milked once a day for 3 consecutive days. Furthermore, the entire herd was milked twice a day, in the morning and in the evening, for the same 3 days collecting 12 milk mass samples.

### 2.4 Sample preparation

The alk profile evaluation was carried out on the herbage samples (N = 48), on the unpasteurized milk samples (48 individual ones and 12 masses) and on the mixed concentrate used for feed supplement.

According to Nardin et al. (2023), the herbage samples were stored at −18°C; then, an aliquot of 2.5 g of homogenized sample (particle diameter of roughly < 2 mm) was weighed in 50‐mL polyethylene falcon tubes (Sartorius AG) and extracted, adding 20 mL of extraction solution (H_2_O/MeOH/FA; 49.5:49.5:1 by vol). The mixture was sonicated for 10 min (50KHz, LBS1 6Lt, FALC Instruments), subjected to vertical shaking for 12 h at 20 g (Rotoshake 24/16; Gerhardt GmbH & Co. KG), and once again sonicated for 10 min. The methanolic extract was separated with centrifugation (10 min at 2481 *g*; IEC CL31 Multispeed; Thermo Scientific), filtered with a 0.45‐µm cellulose filter cartridge (Sartorius AG), diluted twice with a H_2_O/MeOH solution (50:50 by vol) and injected in HPLC‐HQOMS (10 µL).

Adapting the methods proposed by Lamp et al. (2021) and Engel et al. (2022), a homogeneous aliquot of 5 g of milk samples, stored at −18°C, was added to 2 mL of extraction solution (H_2_O/MeOH/FA; 40:40:20 by vol) in polyethylene 50‐mL falcon tubes and sonicated for 15 min. Then, 1 mL hexane was added, the samples were shaken for 10 min, and then the two phases were separated after centrifugation (10 min at 2481 *g*). The hexane phase was removed, and the water layer was filtered with a 0.45‐µm PVDF filter cartridge, diluted twice with H_2_O and injected (30 µL).

For mixed concentrate an aliquot of 2.5 g of homogenized sample (particles diameter roughly < 2 mm) was directly weighed in polyethylene 50‐mL falcon tubes and extracted adding 20 mL of extraction solution (H_2_O/MeOH/FA; 49.5:49.5:1 by vol). The mixture was sonicated for 10 min (LBS1 6Lt; FALC Instruments), subjected to vertical shaking for 12 h at 20 *g* (Rotoshake 24/16; Gerhardt GmbH & Co. KG), and once again sonicated for 10 min. The methanolic extract was separated with centrifugation (10 min at 2481 *g*), filtered with a 0.45‐µm cellulose filter cartridge, diluted twice with a H_2_O/MeOH solution (50:50 by vol) and injected (15 µL).

### 2.5 Chromatographic separation and mass spectrometry detection

According to the methodology suggested by Nardin et al. (2016, 2018), the analysis was carried out using a Thermo Ultimate R3000 UHPLC linked to a hybrid quadrupole‐orbitrap mass spectrometer (Q‐Exactive), equipped with a heated electrospray source (HESI‐II; Thermo Scientific). For online cleanup, a Raptor Biphenyl column (3 mm × 150 mm, 2.7 m particle size; Restek) and a SolEx HRP SPE cartridge (2.1 mm 20 mm, 12–14 m; ThermoFisher) were utilized. Alks were extracted using a binary phase gradient of 0.1% FA with 5 mM ammonium acetate in water and 0.1% FA with 5 mM ammonium acetate in MeOH/ACN 95:5 by vol. Mass spectra were acquired in positive ion mode through a full MS‐data‐dependent MS/MS experiment (full MS–dd MS/MS). Thermo Scientific Dionex Chromeleon 7.2 Chromatography Data System software was used for instrument control and data processing and evaluation.

### 2.6 Targeted and suspect screening studies

Alks were identified using the exported chromatograms (EICs) that corresponded to the protonated molecules [M+H]^+^. For a successful identification, a mass error of less than 5 ppm (SANTE/2020/12830, Rev.1) was set. For analyte quantification, external solvent calibration curves of the 41 standards with concentrations ranging from the limit of quantification (LOQ) to 500 µg L^−1^ were used (Table 1). The quantification was performed from a five‐point calibration curve allowing a regression coefficient (*R* ^2^) of at least 0.99 included in the linearity range. The instrumental limit of detection (LOD) was estimated as three standard deviations of 10 replicated blank (solvent extraction solution) samples according to EURACHEM (2024), and similarly, the LOQ was estimated as 10 standard deviations of the same replicates. For the different matrices, LOD and LOQ were then recalculated based on the dilutions/concentrations of the sample preparation.

**TABLE 1:** Validation parameters for the 41 alkaloid (alk) analytical standards.

			Herb (µg kg^−1^)		Milk (µg kg^−1^)		Animal feed (µg kg^−1^)		
Compound	RT (min)	Accurate mass [M+H]^+^ (Δ*m/z*) (*m/z*; ppm)	LOD	LOQ	LOD	LOQ	LOD	LOQ	*R* ^2^
Nicotine	4.8	163.1232 (1.3)	24.0	80.0	0.41	1.35	15.9	53.0	0.990
Monocrotaline	5.3	326.1589 (0.5)	0.10	0.30	0.001	0.005	0.05	0.18	0.998
Lycopsamine	7.1	300.1809 (1.3)	0.20	0.60	0.003	0.01	0.13	0.42	0.998
Coniine	8.6	128.1429 (3.1)	0.30	1.10	0.01	0.02	0.22	0.75	0.998
Erucifoline	8.8	350.1599 (0.9)	0.10	0.40	0.002	0.01	0.07	0.27	0.998
Senecionine N‐oxide	9.2	352.1751 (1)	0.10	0.30	0.001	0.005	0.05	0.18	0.997
Gramine	9.6	175.1225 (2.3)	0.10	0.30	0.001	0.004	0.05	0.17	0.998
Theobromine/theophylline	9.6	181.0725 (2.8)	4.50	15.0	0.08	0.25	2.97	9.86	0.999
Scopolamine	10.6	304.1539 (1.3)	0.30	1.00	0.01	0.02	0.21	0.69	0.996
Jacobine‐N‐oxide	10.9	368.1715 (3.3)	0.50	1.50	0.01	0.03	0.30	1.00	0.996
Erucifoline‐N‐oxide	11.1	366.1548 (0.3)	0.60	1.90	0.01	0.03	0.37	1.25	0.996
Heliotrine	11.1	314.196 (0.3)	0.10	0.20	0.001	0.004	0.04	0.15	0.998
Retrorsine	11.2	352.1755 (0.3)	0.40	1.30	0.01	0.02	0.27	0.88	0.996
Seneciphylline	12.0	334.1655 (1.8)	0.20	0.90	0.00	0.02	0.15	0.59	0.996
Retrorsine N‐oxide	12.5	368.1708 (1.4)	0.70	2.30	0.01	0.04	0.46	1.52	0.996
Senecionine/senecivernine	13.2	336.1801 (1.3)	0.20	0.70	0.004	0.01	0.15	0.49	0.993
Hyoscyamine/atropine	13.3	290.1755 (1.5)	0.20	0.80	0.004	0.01	0.16	0.54	0.994
Echimidine	14.3	398.2172 (0.3)	0.10	0.40	0.002	0.01	0.08	0.27	0.999
Senkirkin	14.5	366.1918 (1.9)	1.10	3.60	0.02	0.06	0.70	2.35	0.998
Jacobine	14.9	352.1762 (2.3)	0.50	1.50	0.01	0.03	0.30	1.01	0.997
Caffeine	15.1	195.0881 (2.1)	1.50	4.90	0.02	0.08	0.98	3.24	0.998
Lasiocarpine	16.5	412.2325 (1)	0.20	0.70	0.003	0.01	0.13	0.43	0.998
Striknine	17.2	335.1751 (0.9)	0.90	3.00	0.02	0.05	0.63	1.97	0.993
Harmaline	17.3	215.1185 (2.9)	5.60	19.0	0.09	0.31	3.70	12.30	0.996
Sipeimine	18.0	430.3311 (0.9)	1.90	6.30	0.03	0.11	1.25	4.16	0.996
Quinine	18.2	325.1909 (0.5)	3.10	10.0	0.05	0.18	2.07	6.89	0.995
Quinidine	18.2	325.1901 (2.9)	4.60	16.0	0.08	0.26	3.07	10.28	0.998
Veratramine	20.7	410.3069 (3.9)	0.30	0.90	0.005	0.02	0.19	0.63	0.995
Alpha‐solasonine	20.7	884.5008 (0.7)	0.20	0.60	0.003	0.01	0.11	0.37	1000
Jervine	20.8	426.3005 (0.7)	0.40	1.40	0.01	0.02	0.28	0.91	0.995
Alpha‐solamargine	21.1	868.5056 (0.5)	4.40	15.0	0.07	0.25	2.89	9.65	0.999
Protoveratrine A	21.2	794.4322 (0.1)	17.0	57.0	0.29	0.96	11.34	37.84	0.996
Alpha‐solanine	22.1	868.5059 (1)	0.20	0.70	0.004	0.01	0.15	0.48	0.998
Veratridine	22.2	674.3521 (1.9)	2.00	6.80	0.03	0.12	1.36	4.52	0.998
Solasodine	24.5	414.3375 (2)	1.00	3.50	0.02	0.06	0.69	2.29	0.993
Aconitine	24.5	646.3229 (1.2)	2.00	6.60	0.03	0.11	1.31	4.37	0.999
Tomatidine/tomatine	25.4	416.3535^a^ (2.9)	10.0	32.0	0.16	0.54	6.35	21.20	0.991

Abbreviations: LOD, limit of detection; LOQ, limit of quantitation; RT, retention time; Δ *m/z* (ppm), error of accurate mass respect to exact mass.

^a^ Tomatine [M+H‐C_23_H_38_O_19_]^+^.

In order to extend the study of alk transfer from herbs to milk, a suspect screening approach was adopted, according to previous studies performed by Larcher and Nardin (2019). Retention times (RTs) and the confirmation fragments of 93 among aglycon alks and their glycosidic precursors are reported in Table 2. The study of suspected alks was limited to compounds providing a sufficient detectable response (area > 300 area units). The presence of isomers is very common in this chemical group; in fact, situations have arisen where multiple peaks were present in the EIC. Since it was impossible to assign a name to just one of these peaks, the peaks were identified with compound@RT.

**TABLE 2:** Chromatographic parameters of alkaloids and glycosidic alkaloids tentatively identified with the suspect screening method.

Alkaloid	RT (min)	Accurate mass [M+H]^+^ (*m/z*)	Experimental fragments (*m/z*)	Alkaloid	RT (min)	Accurate mass [M+H]^+^ (*m/z*)	Fragments (*m/z*)
Retronecine^a^	2.5	156.1016 (−1.9)	138.0907; 112.0772	8‐Methyl‐10‐phenyllobelidiol/norlelobanidine	16.1	278.211 (1.8)	156.1378; 138.1282
Betonicine	3.0	160.0962 (2.4)	72.0812; 142.0857	Lobinanidine, isolobinanidine, beta‐lobinanidine	16.2	290.2108 (2.4)	168.1375; 96.0807
Valerine	3.2	158.1175 (1.8)	112.0760	19Z‐16‐epi‐Voacarpine/16‐epi‐Voacarpine	16.3	369.1802 (1.9)	321.1591; 291.1478
3‐Acetyltropine	3.8	184.1329 (1.6)	166.0853	Cinchonanine C	16.3	311.1748 (1.9)	293.1641; 239.1174
3,6‐Diacetyltropine	3.9	226.1433 (2.2)	110.0598; 164.0715	Mesaconine	16.3	486.2689 (1.9)	70.06558; 130.08618
Cathinine	5.3	150.0911 (1.5)	132.1018; 135.0677	Lelobanidine I/II hexoside 3	16.4	454.2795 (0.9)	98.0966; 170.1537; 292.2264
Ginkotoxin	5.5	184.0965 (1.7)	152.0719; 134.05962	Cheilanthifoline hexoside 2	16.5	488.192 (−1.0)	326.1375; 178.0858
8‐Ethylnorlobelol	5.6	158.1536 (1.9)	84.9598; 140.1424; 98.9750	Lelobanidine I/II hexoside‐hexoside	16.6	616.3337 (−1.5)	292.2259; 170.1532
Indicain	5.8	162.0912 (0.7)	144.0807; 70.0657	8‐Methyl‐10‐phenyllobelidiol/norlelobanidine	16.6	278.2109 (2.2)	156.1378; 138.1269
Intermedine^a^	7.1	300.1807 (0.3)	138.0905; 94.0658	Caryachine hexoside 2	16.6	488.1916 (−0.2)	326.1382; 295.0961
Norallosedamine pentoside 1	7.5	338.1958 (1.2)	206.1174	Fumarophycine	16.6	398.1591 (1.8)	338.1377; 277.0851
Norallosedamine pentoside 2	8.1	338.1958 (1.2)	206.1173	Mesaconine	16.6	486.2696 (0.5)	70.06584; 130.08654
Intermedine N‐oxide^a^	8.3	316.1751 (−1.3)	138.0907	Lobinine/isolobinine	16.8	288.1952 (2.1)	168.1378; 96.0808
Cinchonanine F deoxyhexoside 1	8.4	473.2643 (0.6)	327.2064; 160.0754	Lelobanidine I/lelobanidine II	16.9	292.2263 (2.4)	170.1540; 202.1580; 98.0961
Lycopsamine N‐oxide^a^	8.7	316.1757 (0.6)	138.0904	Aconine	17.2	500.2849 (1.0)	84.06105; 144.1016
Valerianine	9.0	178.1225 (0.8)	146.0960	Cevadine	17.2	592.3474 (1.0)	574.3358; 556.3259
8,10‐Diethyllobelidiol hexoside‐hexoside 1	9.8	568.3329 (−0.2)	244.2267; 226.2162; 406.2802	Cheilanthifoline	17.2	326.1384 (0.8)	178.0833; 151.0724
Cheilanthifoline hexoside 1	9.9	488.1908 (1.4)	326.1374; 178.0857	19Z‐16‐epi‐Voacarpine/16‐epi‐voacarpine	17.4	369.1814 (1.4)	321.1591; 291.1478
Ridelline^a^	9.9	350.1595 (−0.9)	120.0807	Lelobanidine I/lelobanidine II	17.4	292.2271 (2.7)	170.1538; 202.1580; 98.0959
Seneciphylline hexoside	10.1	496.2173 (0.8)	120.0808; 334.1646	Lobinanidine, isolobinanidine, beta‐lobinanidine	17.4	290.2109 (2.1)	168.1380; 96.0809
Valerianine	10.1	178.1223 (1.6)	146.0960	Aconine	17.5	500.2836 (3.5)	84.06099; 144.1016
Retrorsine hexoside 1	10.2	514.2272 (1.9)	120.0806; 352.1743	N‐Methyllaurotetanine	17.5	342.1695 (1.3)	280.1089; 296.1037
Anisodamine	10.4	306.169 (3.3)	140.1060; 122.0965	Protopine	17.5	354.1332 (1.1)	149.0595; 189.0782
Retrorsine hexoside 2	10.5	514.2275 (1.4)	120.0806; 352.1744	Lobinanidine, isolobinanidine, beta‐lobinanidine	17.6	290.2115 (2.1)	168.1380; 96.0809
Senecionine hexoside	10.6	498.2333 (0.2)	336.1795; 120.0807	Caryachine	17.8	326.1382 (1.5)	295.0885; 263.0717
Fumarophycine hexoside‐hexoside	10.8	722.2641 (1.8)	338.1375; 398.1583	cis/trans lobeline hexoside 1	17.8	500.2638 (1)	96.0810; 338.2108
Retrorsine hexoside 3	10.9	514.2272 (1.9)	120.0806; 352.1738	Apohyoscyamine	17.9	272.1645 (2.6)	–
8‐Methyl‐10‐phenyllobelidiol/norlelobanidine hexoside‐hexoside 1	11.6	602.3173 (−0.3)	156.1382; 278.2110; 440.2621	Cevadine	18.1	592.3471 (1.5)	574.3346; 556.3260
Seneciphylline N‐oxide^a^	11.7	350.1599 (0.3)	322.1646; 119.0734	Fumaricine	18.3	370.1641 (2.2)	309.0755; 352.1535
Cinchonanine F deoxyhexoside 2	11.8	473.2645 (0.2)	327.2062; 160.0755	Fumarilin	18.3	352.117 (2.5)	337.1299; 322.1435
Gelsemine	11.9	323.1743 (3.5)	70.0654; 236.1065	o‐Methylcaryachine	18.3	340.1538 (1.5)	295.0951; 263.0702
Cinchonanine F	12.0	327.206 (2.2)	309.1948; 160.0753	Escholtzine	18.5	324.1227 (0.9)	293.0803; 188.07034
Fumarophycine hexoside	12.0	560.2125 (0.2)	338.1361; 398.1598	Gelsempervine‐B/gelsempervine‐D	18.5	425.2071 (1.9)	172.0752; 180.1009; 158.0608
8,10‐Diethyllobelidiol	12.1	244.2262 (3.7)	226.2176; 170.1536	cis/trans Lobelanidine hexoside‐hexoside1	18.9	664.3334 (−1.1)	202.1584; 340.2266; 502.2777
Retrorsine deoxy hexoside	12.2	498.2325 (1.6)	120.0805; 352.1737	Gelsempervine‐B/gelsempervine‐D	18.9	425.2071 (1.9)	172.0752; 180.1009; 158.0608
8,10‐Diethyllobelidiol hexoside	12.3	406.2795 (1.0)	244.2268; 226.2168	Parfumidine	18.9	368.1484 (2.3)	353.1243
8‐Methyl‐10‐phenyllobelidiol/norlelobanidine hexoside‐pentoside	12.5	572.3063 (−0.5)	156.1384; 278.2110	Cinchonanine F	19.0	327.2059 (2.6)	309.1953; 160.0754
Cheilanthifoline hexoside‐hexoside	12.5	650.2428 (0.3)	488.1892; 326.1382; 243.0886	cis/trans Lobeline hexoside2	19.1	500.2639 (0.8)	96.0810; 338.2108
8,10‐Diethyllobelidiol hexoside‐hexoside 2	12.8	568.3333 (−0.9)	244.2267; 226.2162; 406.2802	cis/trans Lobelanidine hexoside	19.2	502.2797 (0.4)	202.1588; 340.2265
Hyoscyamine hexoside	12.8	452.2277 (0.4)	124.1121; 290.1731	Caryachine	19.3	326.1382 (1.5)	295.0885; 263.0717
Cinchonanine F hexoside	12.9	489.2598 (−0.6)	327.2062; 160.0758	Methyl‐fumarophycine	19.3	412.1748 (1.8)	277.0855; 249.0906
Actinidine	13.0	148.1118 (2.1)	120.0196; 130.0861	Cinchonanine B	19.5	309.1592 (1.7)	136.11162; 121.0885
8,10‐Diethyllobelidiol	13.1	244.2264 (2.9)	226.2148; 170.1538	Gelsemicine	19.6	359.1965 (2.2)	328.1759; 299.1401
Caryachine hexoside 1	13.2	488.1911 (0.8)	326.1381; 295.0959	Parfumidine	19.7	368.1486 (1.7)	353.1243
8,10‐Diethyllobelidiol pentoside	13.4	376.2693 (0.3)	244.2267; 226.2178	cis/trans Lobelanidine hexoside‐hexoside2	19.8	664.3333 (−0.9)	202.1536; 340.2260
8‐Methyl‐10‐phenyllobelidiol/norlelobanidine hexoside‐hexoside 2	13.9	602.3162 (1.5)	156.1383; 278.2110;	cis/trans Lobeline hexoside3	19.8	500.2635 (1.6)	96.0810; 338.2108
Norharmane	14.2	169.0758 (1.2)	81.0722; 109.0647	Norallosedamine	19.8	206.1539 (1.5)	122.0964; 105.0699
8‐Methyl‐10‐phenyllobelidiol/norlelobanidine hexoside	14.3	440.264 (0.7)	156.1381; 278.2110	Lobinine/Isolobinine	20.0	288.1958 (2.1)	168.1378; 93.0808
Harmol	14.4	199.0862 (1.9)	181.1111; 135.1172	cis‐Lobelanidine/*trans*‐lobelanidine	20.2	340.2271 (2.1)	202.1584; 218.15441
Echimidine N‐oxide^a^	14.5	414.2326 (−0.7)	254.1385; 352.1738	cis‐Lobeline*/trans‐*lobeline	20.2	338.2115 (2.4)	216.1361; 96.0808
Caryachine	14.5	326.1382 (1.5)	311.1134; 295.0885	cis‐Lobeline*/trans‐*lobeline	20.5	338.2115 (2.4)	216.1386; 96.0808
Cinchonanine C	14.8	311.1747 (2.4)	293.1642; 239.1173	cis‐Lobelanine*/trans‐*lobelanine	20.7	336.1958 (1.8)	96.0814; 216.1378
Gelsempervine‐A/gelsempervine‐C	15.3	383.1958 (1.8)	180.1011; 321.1599; 166.0864	cis‐Lobelanidine/*trans*‐lobelanidine	20.9	340.2271 (2.4)	218.1544; 202.1583
8‐Methyl‐10‐phenyllobelidiol/norlelobanidine	15.5	278.2108 (2.5)	156.1376; 138.1270	Norlobelanine	20.9	322.1802 (2.5)	202.1223; 82.0657; 171.1392
Harmane	15.5	183.0914 (1.8)	81.0706; 105.0706	cis‐Lobelanine*/trans‐*lobelanine	21.2	336.1958 (0.6)	96.0813; 216.1380
Gelsempervine‐A/gelsempervine‐C	15.6	383.1958 (1.8)	180.1015; 321.1600; 166.0864	Corydamine	22.4	351.1323 (4.6)	293.1339; 336.1199
Lelobanidine I/II hexoside1	15.6	454.2793 (1.3)	170.1536; 292.2265	Dihydropiperlonguminine	22.6	276.159 (1.4)	135.0443
Cinchonanine E	15.7	295.1801 (1.5)	277.1693	Piperlonguminine	23.0	274.1433 (1.8)	201.0541; 135.0438
Norallosedamine pentoside 3	15.8	338.1957 (1.5)	206.1170	Cinchonanine B	23.4	309.1591 (2.0)	136.11162; 121.0885
Harmane	15.9	183.0914 (1.8)	81.0706; 105.0706	Piperanine	25.1	288.1587 (2.5)	203.1059; 135.0438
Cinchonanine G	16.0	329.1854 (1.9)	309.1955; 160.0755	Arborinine	25.5	286.1069 (1.7)	271.0834; 253.0729
Lelobanidine I/II hexoside2	16.0	454.2798 (0.2)	98.0966; 170.1537; 292.2267	Piperine	25.5	286.1431 (2.4)	201.0542; 135.0438
o‐Methylcaryachine	16.0	340.1537 (1.9)	309.1092; 188.0704				

Abbreviations: Δ*m/z* (ppm), accurate mass error; RT, retention time.

^a^ Alkaloids confirmed with subsequently purchased standards.

### 2.7 Statistical analysis

Univariate and multivariate analyses were performed using the XLSTAT 2023 (Addinsoft) software. Statistical processing was carried out on concentration values for targeted alks, while the ionization intensity expressed as peak area was used for suspected alks. Kruskal Wallis test for an unequal number of samples (*p* < 0.05) was performed to identify significant differences in the alk content of herbs collected in the two distinct pastures and milk samples collected from cows that grazed in the two same pastures. A principal component analysis (PCA) was carried out to evaluate the relationships between alks and the milk samples coming from the two different pastures. Partial least squares—discriminant analysis (PLS‐DA) with a Jackknife (LOO) cross‐validation was used to test the efficacy of alk profiles in discriminating the different pastures and different origins of milk samples. The optimum number of PLS components was estimated using full cross‐validation. Classification performance was assessed in the validation phase in terms of sensitivity, specificity, and accuracy.

## 3 RESULTS AND DISCUSSION

### 3.1 Characterization of the potential alk intake of cows

A different herbage intake composition was observed in the two experimental pastures, due to the different botanical composition (Table 3). Particularly in the PO pasture, families not belonging to the top 10 represented 2.36% of available herbage but reached almost 20% of the selected herbage mass, with a selectivity index (ratio between family percentages in selected and available mass) equal to 8.4. Apart from this group, the most selected families were Fabaceae (selectivity index = 4.2), Rosaceae (3.6), and Cariophyllaceae (3.4) in PO pasture, and Apiaceae (3.0), Plantaginaceae (2.8), and again Fabaceae (1.6) in SE pasture. Other families were instead avoided or under‐grazed by dairy cows, particularly Liliaceae (selectivity index = 0.1 in PO pasture), Asteraceae (0.2 and 0.4 in Se and PO, respectively), and Hypericaceae (0.3 and 0.8 in Se and PO, respectively).

**TABLE 3:** Contribution (expressed as a dry matter percentage) of the main botanical families to the available and the selected herbage masses of two experimental alpine pastures (*Poion alpinae*, PO; *Seslerion caeruleae*, SE).

	PO			SE	
Botanical families	Available	Selected	Botanical families	Available	Selected
Poaceae^a^	66.5	41.5	Poaceae^a^	61.8	57.5
Ranuncolaceae	7.37	4.29	Asteraceae	8.47	2.10
Liliaceae	5.11	0.48	Fabaceae	5.36	8.63
Asteraceae	3.53	1.36	Rosaceae	3.33	3.66
Poligonaceae	3.35	1.27	Plantaginaceae	3.17	8.95
Fabaceae	2.82	11.8	Lamiaceae	2.93	2.45
Hypericaceae	2.47	2.00	Hypericaceae	2.85	0.92
Rosaceae	2.38	8.45	Scrophulariaceae	2.22	2.08
Scrophulariaceae	2.17	2.37	Poligonaceae	1.66	1.54
Cariophyllaceae	1.96	6.59	Ranuncolaceae	1.64	0.92
Other families	2.36	19.9	Apiaceae	1.59	4.83
			Other families	4.98	6.42

^a^ *Poaceae* includes other families belonging to the Poales order, such as Cyperaceae and Juncaeae.

The results of targeted alk analysis of the 48 manual herbage samples are reported in Table 4. Only four alks showed content above the LOD in at least one sample. Gramine (indole alk; Ind) and veratramine (steroidal alk, Str) were the most frequently found alks. Gramine was quantified with a concentration of 45 and 86 µg kg^−1^ in two PO samples and of 11 and 65 µg kg^−1^ in other two SE samples, while veratramine was present in four samples only collected in PO pasture, with a concentration between the 20 and 357 µg kg^−1^. This alk was found only in *Phleum r*., a Poaceae detected in all botanical assessments of PO pasture, where its coverage surface averaged 14.6% (Table 3). Lycopsamine was detected in only one PO sample (8.5 µg kg^−1^) and in one SE sample (3.4 µg kg^−1^). Finally, veratridine (Str) was found in one of the samples collected in SE at 15.6 µg kg^−1^, probably carried by a sporadic unidentified herb. The lack of erucifoline, heliotrine, and seniciphylline detection in the hand‐plucked samples is likely a consequence of the selective behavior of cows, avoiding plants (i.e. *Hypericum m*. and *Potentilla c*.) containing unpalatable substances (Cortinovis & Caloni, 2015).

**TABLE 4:** Minimum, median, and maximum values for alk content (µg kg^−1^) in the herbage selected in experimental pastures (*Poion alpinae*, PO; *Seslerion caeruleae*, SE).

	Herbage from PO				Herbage from SE			
Alkaloid	Samples (N > LOD)	Min	Med	Max	Samples (N > LOD)	Min	Med	Max
Gramine	4	<0.1	<0.1	86	2	<0.1	<0.1	64
Lycopsamine	1	<0.2	<0.2	8.5	1	<0.1	<0.1	3.4
Veratramine	4	<0.3	<0.3	354	0	<0.3	<0.3	<0.3
Veratridine	0	<0.2	<0.2	<0.2	1	<0.2	<0.2	15

Abbreviations: LOD, limit of detection; LOQ, limit of quantitation; Max, maximum; Med, median; Min, minimum.

With regard to the occurrence of suspected alks in the selected herbage samples, 66 different alks were detected (Table S2). Twenty‐five corresponded to piperidine alks (Pprs) which were present in the 94% of the herb samples (92% in PO, 100% in SE). The majority of these alks were present in mono‐ or di‐glycosylated form. Note that 8,10‐diethyllobelidiol pentoside, 8,10‐diethyllobelidiol@13.1, 8‐ethylnorlobelol, cis/trans lobelanidine hexoside, cis/trans lobelanidine hexoside‐hexoside@18.9, and cis/trans lobelanidine hexoside‐hexoside@19.7 were present only in SE herb mixes, while cis‐lobelanidine/trans‐lobelanidine@20 and piperanine only in PO samples. Fourteen alks belonged to isoquinoline alks (Iqns) which were identified in the 67% of herb mixes (50% in PO, 88% SE), also in this case a high presence of glycosylates was found. As far as the other alk groups are concerned, PO turned out to be numerically richer than SE in acridone alks (Acds; 8% and 4% respectively), while SE was found to be richer than PO in terpene (Trns; 54% and 42%), quinolines (Qnls; 46% and 25%), Strs (25% and 17%), Trps (17% only in SE), and pyridines (Pyrs; 8% only in SE).

### 3.2 Discrimination of the potential alk intake of cows grazing on different alpine pastures

Considering the alk profiles of the herbal mixes sampled in the two different pastures, Kruskal–Wallis test highlighted the following significant differences: PO was significantly richer (*p* < 0.05) in veratramine, while cis/trans lobelanidine hexoside‐hexoside@18.9, cis/trans lobelanidine hexoside, cis/trans lobelanidine hexoside‐hexoside@19.7, and norallosedamine pentoside@15.8 were more abundant in SE.

**FIGURE 1:**
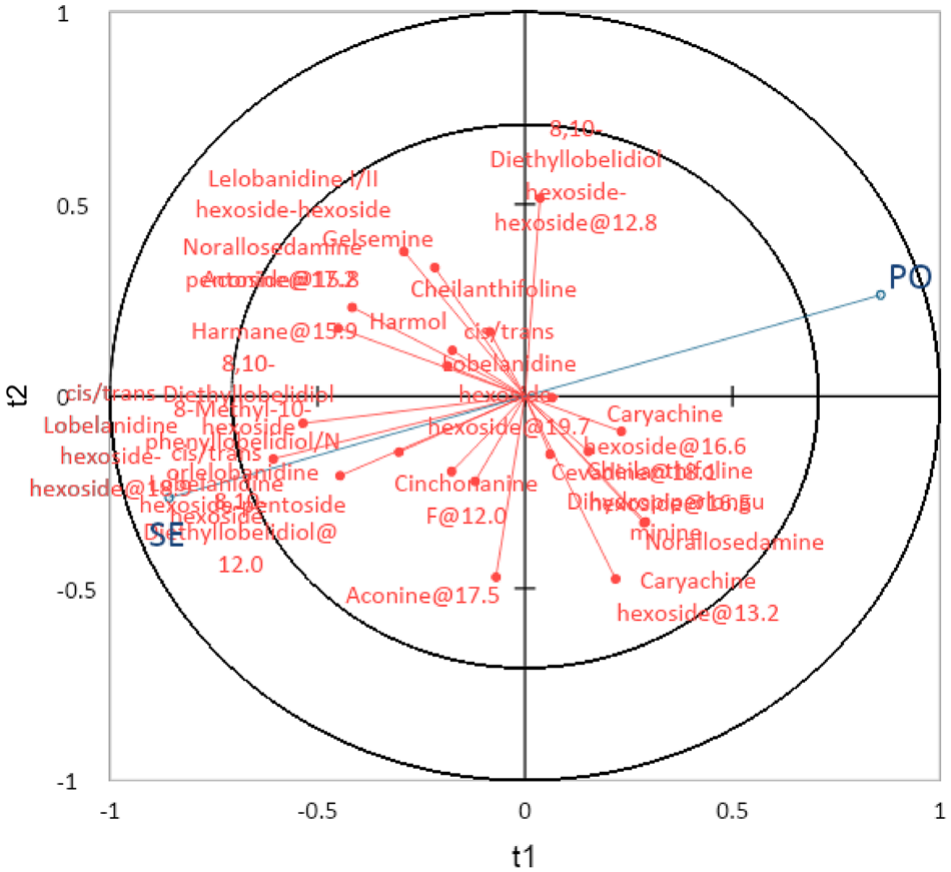
Correlation loadings in a bi‐factor partial least squares—discriminant analysis model (*R* ^2^_C_ = 0.98) for the classification of cow diet samples (hand plucked) according to the type of grazed pasture (blue dots, Y‐matrix, PO = *Poion alpinae*; SE = *Seslerion caeruleae*), based on their alkaloid profile (red dots, X‐matrix).

**FIGURE 2:**
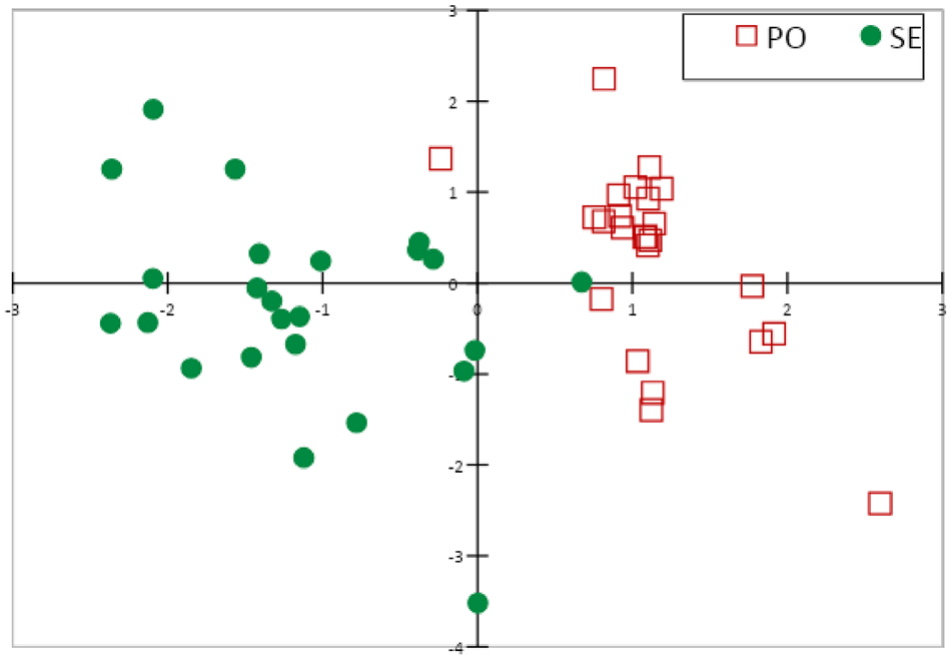
Score‐plot of cow diet samples (hand‐plucked) from the partial least squares—discriminant analysis model of grazed pasture type (red square, PO = *Poion alpinae*; green dots, SE = *Seslerion caeruleae*) classification based on their alkaloid profile.

PLS‐DA was used to verify the viability of a model able to discriminate between the potential alk intake of dairy cows grazing on the two different alpine pastures. The details of the model are summarized in Figure 1, while score plot results of the model are shown in Figure 2.

After having considered all the available alks, the proposed bi‐factorial model considered only those present in at least six samples. Those with the highest variable importance in the projection (VIP > 1) were cis/trans lobelanidine hexoside‐hexoside@18.9, cis/trans lobelanidine hexoside, cis/trans lobelanidine hexoside‐hexoside@19.7, norallosedamine pentoside@15.8, aconine@17.2, and 8,10‐diethyllobelidiol@12.0. The performance of the model was satisfying, with total accuracy of 98%. Indeed, none out of 24 samples hand‐plucked in PO pasture and one out of 24 samples selected in SE pasture were incorrectly assigned to the other pasture (Table S3).

### 3.3 Transfer of alk from herbs to milk

**FIGURE 3:**
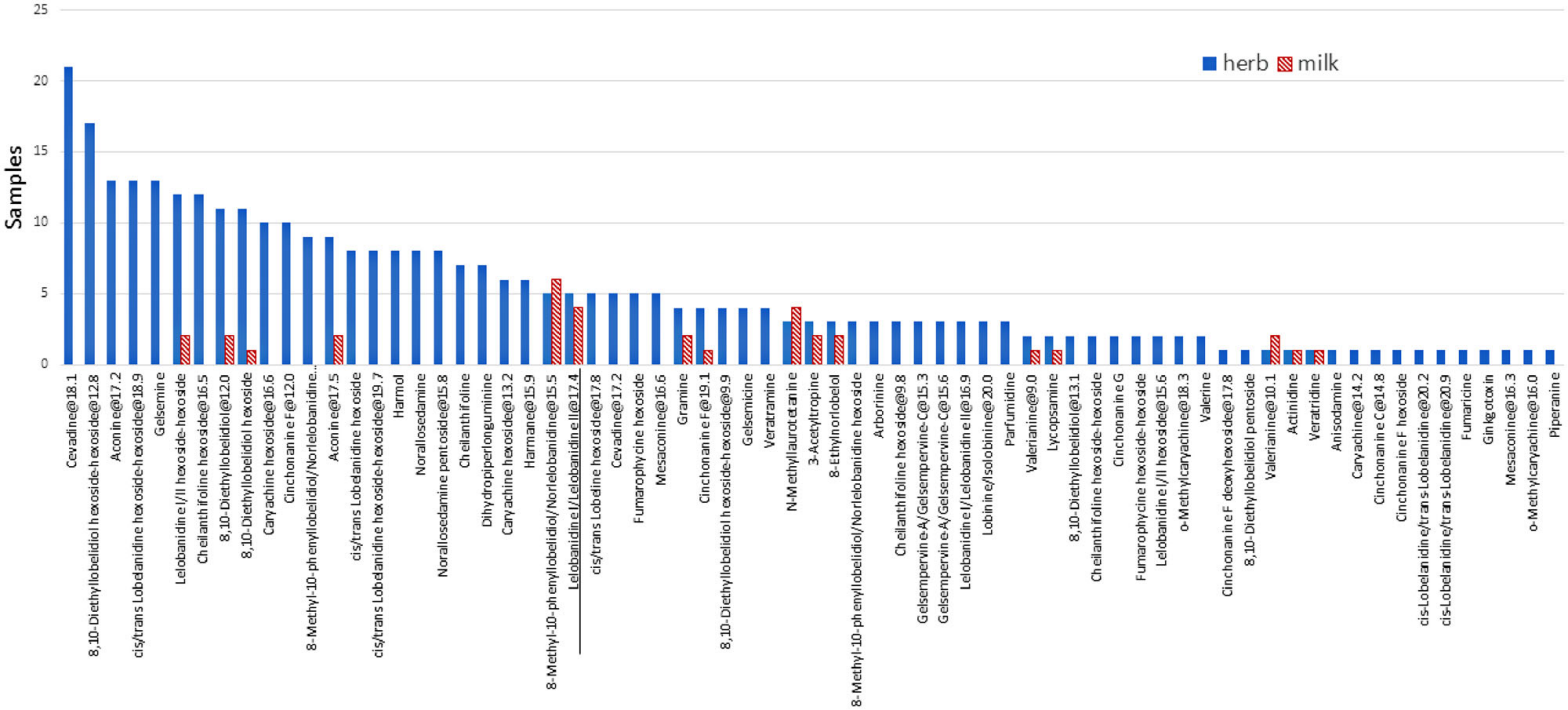
Carry‐over of alkaloids from 48 selected herbages (hand‐plucked) to 48 milk samples collected from the observed cows.

The average milk production data, recorded during 2‐week preliminary periods, are as follows: milk yield (17.4 ± 2.1 kg day^−1^; average ± SD), stage of lactation (148 ± 59.8 days in milk), fat (3.84 ± 0.46%), protein (3.14 ± 0.15%), lactose (4.68 ± 0.14%), and somatic cell count (124,800 ± 117,000 cells mL^−1^). Figure 3 compares the presence (as number of positive samples) of alks in herbages and in milk samples of the two pastures. The alk present also in the mixed concentrate is reported as underlined (lelobanidine I/lelobanidine II@17.4) and was not considered in the subsequent statistical evaluations. Also, indicain was detected in mixed concentrate, but it was never found in herb mixture and neither in milk samples.

Considering the milk samples, a significantly lower number of alks (*n* = 19) were detected in comparison to the herbs. In particular, gramine was quantified in only one milk derived from both pastures (Table 5) at the concentration of 0.38 µg kg^−1^ (PO) and 0.43 µg kg^−1^ (SE). In a PO milk sample, lycopsamine was quantified at 0.1 µg kg^−1^, while in an SE sample, veratridine was detected at 1.6 µg kg^−1^. To establish the actual transfer of alks from herbs to milk is particularly complex, as the cows were not forced to feed on a specific type of herb but were free to graze. Taking the example of cow number 106, one of its herbage samples showed a content of lycopsamine (8.53 µg kg^−1^), and the corresponding milk contained 0.1 µg L^−1^. Considering an estimated milk production of about 20 L day^−1^ cow^−1^ and a daily herbage intake of 60 kg, the average transfer of lycopsamine was assessed to be 0.4%. This confirms what has already been reported by Mulder et al. (2020), who, considering Pyzs, estimated a milk transfer ranging from 0.01% to 1.4% and showed the majority excretion through urine or feces. For gramine, considering cow number 104, which contained 0.37 µg L^−1^ in one collected milk and 4.6 µg kg^−1^ in related herbs, the transfer was assessed to be 2.7%. For veratridine, considering cow 123 with a milk sample with a content of 5.6 µg L^−1^ and a herbal sample of 15.6 µg kg^−1^, the transfer was estimated to be above 12%. No information is reported in the literature for the transfer of Inds and Strs from herbs to milk samples. Regarding Trps, Lamp et al. (2021) estimated a medium transfer of 0.05%. In our milk, Trps have not been quantified, but the data are interesting for understanding that different chemical groups of alks result in different transfers, albeit with limited values.

**TABLE 5:** Minimum, median, and maximum values for alk content (µg kg^−1^) in the individual and masses milk from the two experimental pastures (*Poion alpinae*, PO; *Seslerion caeruleae*, SE).

	Individual milk from PO (N = 24) (µg kg^−1^)				Individual milk from SE (N = 24) (µg kg^−1^)				Milk masses from PO (N = 6) (µg kg^−1^)				Milk masses from SE (N = 6) (µg kg^−1^)			
Alkaloid	Samples (N > LOD)	Min	Med	Max	Samples (N > LOD)	Min	Med	Max	Samples (N > LOD)	Min	Med	Max	Samples (N > LOD)	Min	Med	Max
Gramine	1	<LOD	<LOD	0.38	1	<LOD	<LOD	0.43	0	<LOD	<LOD	<LOD	0	<LOD	<LOD	<LOD
Lycopsamine	1	<LOD	<LOD	0.1	0	<LOD	<LOD	<LOD	1	<LOD	<LOD	0.05	0	<LOD	<LOD	<LOD
Veratridine	0	<LOD	<LOD	<LOD	1	<LOD	<LOD	1.6	0	<LOD	<LOD	<LOD	0	<LOD	<LOD	<LOD

Abbreviations: LOD, limit of detection; LOQ, limit of quantitation; Max, maximum; Med, median; Min, minimum.

Regarding suspected alks, Pprs were detected in the 17% of both PO and SE milk samples. Qnls were found in 17% of milk samples collected from cows that were grazed in PO and 4% in those from SE. Trns in the 8% of both origins, and Trps were found only in the 8% of SE milk collected samples. Different from what was observed in the herbs, glycosylated alks were not identified. This was plausible considering that during digestion in an acidic context, the molecule undergoes hydrolysis.

**FIGURE 4:**
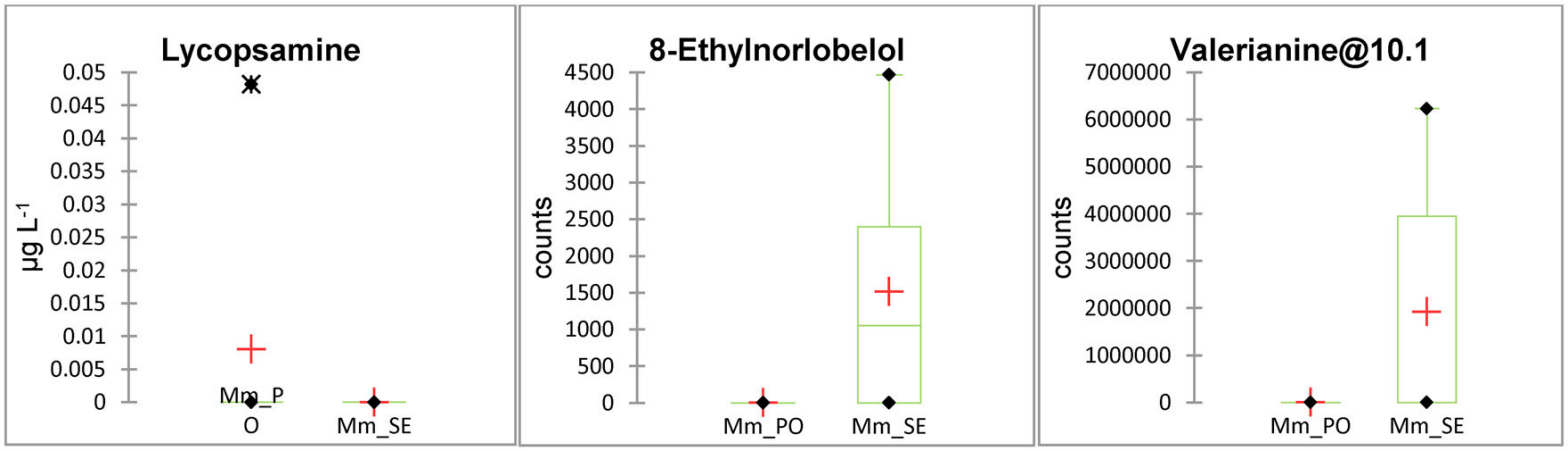
Box plots of alkaloids distributed in the milk masses (Mm_PO = milk masses from *Poion alpinae*; Mm_SE = milk masses from *Seslerion caeruleae*).

In the milk masses, probably due to the dilution as a result of the mixing of the individual milks in the two masses, only three alks were identified above the minimum reference area or above LOD. Lycopsamine at 0.05 µg kg^−1^ was found in one PO milk, 8‐ethylnorlobelol was found in three SE samples, and valerianine@10.1 was found in two SE samples (Figure 4).

### 3.4 Discrimination of alpine milk samples

**FIGURE 5:**
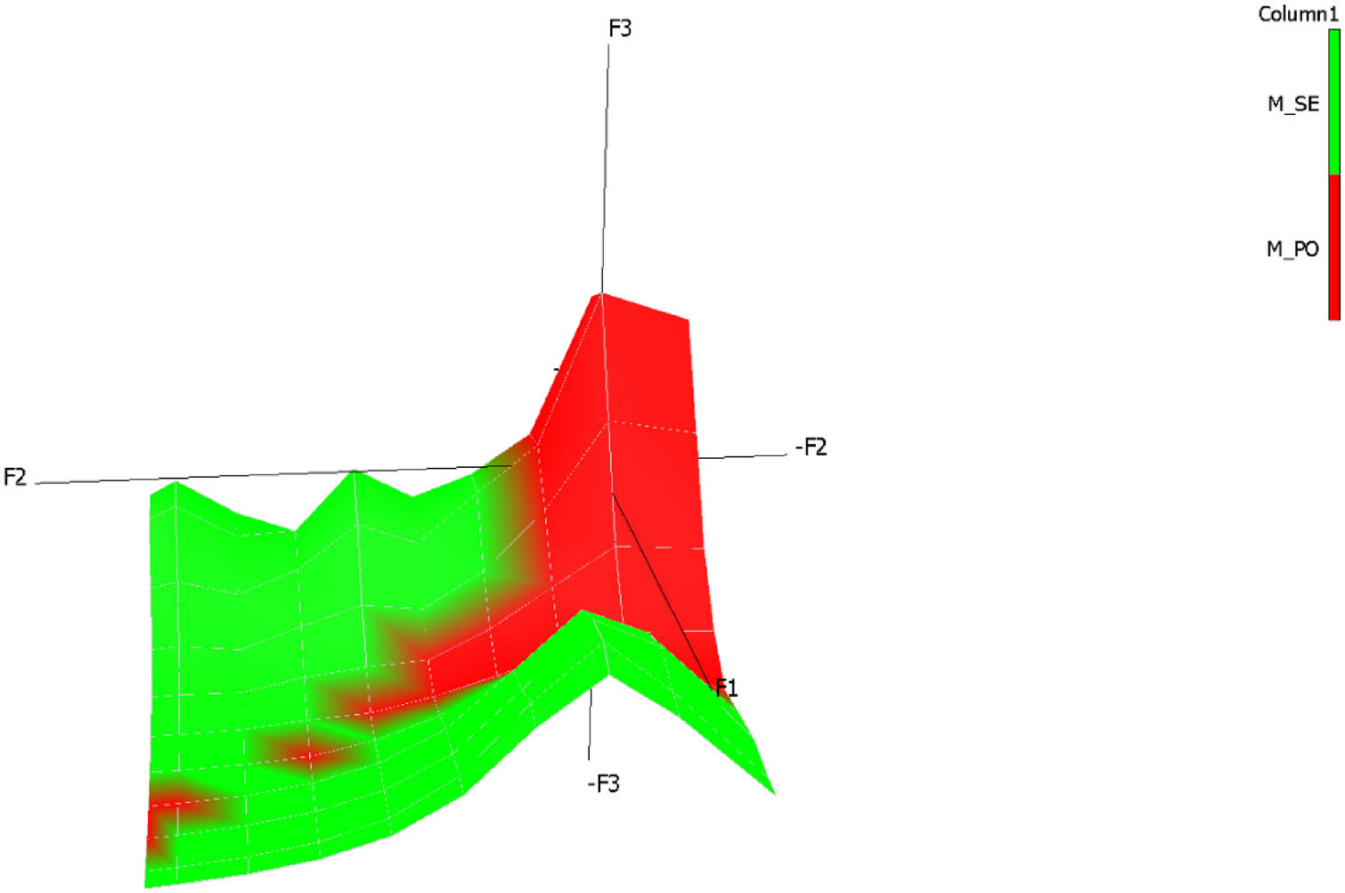
Principal component analysis of milk masses according to the two alpine pastures (red = milk from *Poion alpinae*; green = milk from *Seslerion caeruleae*).

With regard to the single alks present in the individual milk collected in the two different alpine pastures, Kruskal–Wallis test (*p* < 0.05) was performed on non‐normally distributed compounds. Unfortunately, the test indicated no significant differences based on the alks found in the milk samples. The 3D surface plot (Figure 5) showed the distribution of the scores explaining the variance of 55.2%. The milk samples from PO are mainly distributed in the upper part of the graph, while samples from the SE pastures are on the plane. Two distinct groups are observed regarding these milk samples, which could indicate a different diet of the cows themselves in the same pasture. The distinction among the two milk groups (PO and SE) is not clear, but this was expected, considering that two pastures of different altitudes in the same region were still considered, where a good portion of the flora is common (Tables S1 and Table 3).

**FIGURE 6:**
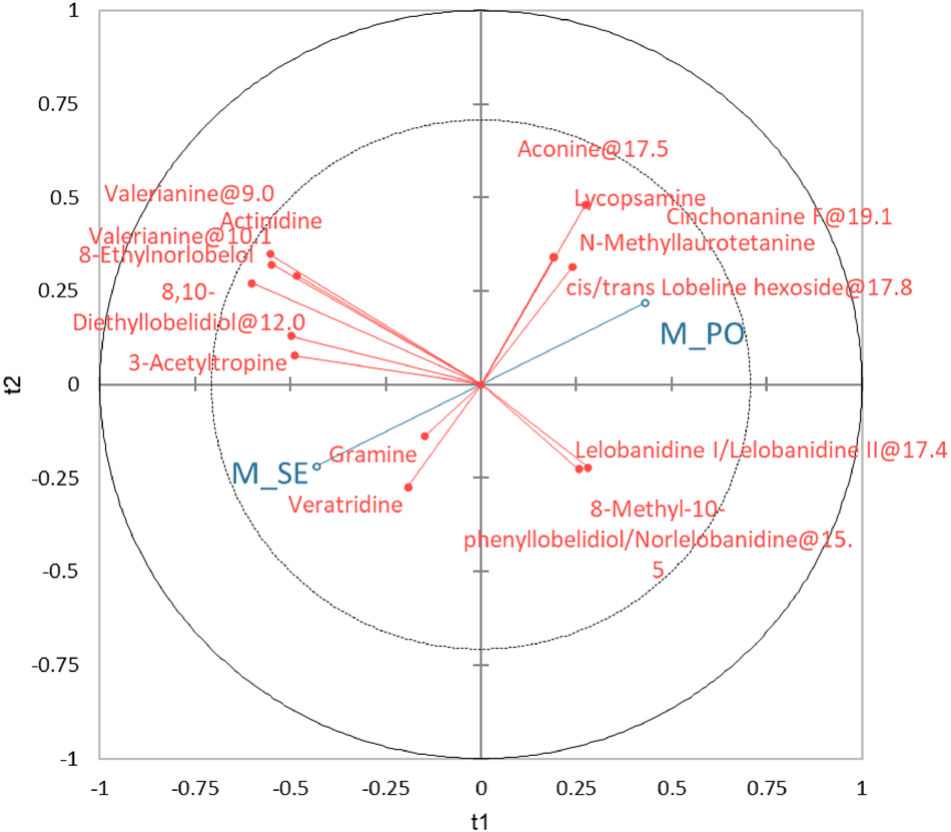
Correlation loadings in a bi‐factor partial least squares—discriminant analysis model (*R* ^2^_C_ = 0.67) for the classification of milk samples according to the type of grazed cow pasture (blue dots, Y‐matrix, M_PO = milk from *Poion alpinae*; M_SE = milk from *Seslerion caeruleae*) based on their alkaloid profile (red dots, X‐matrix).

**FIGURE 7:**
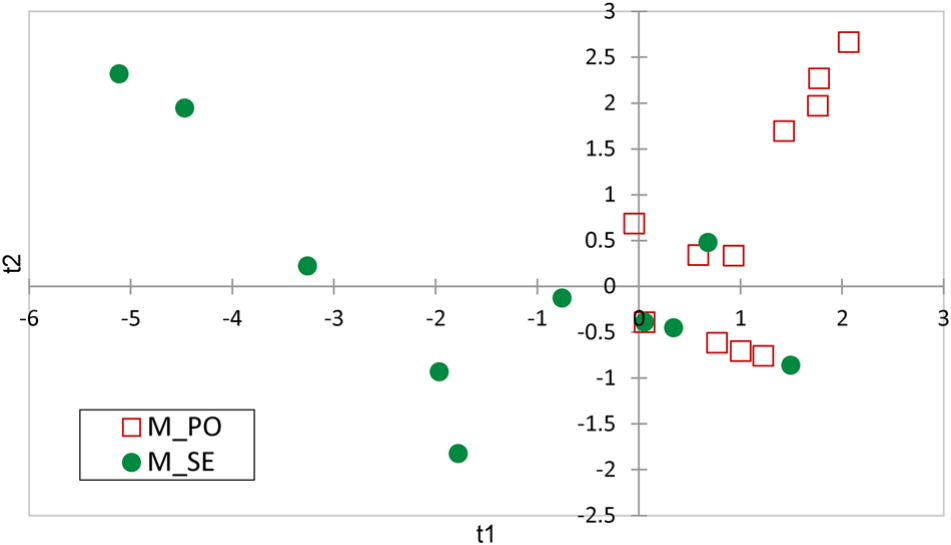
Correlation loadings in a partial least squares—discriminant analysis model for the classification of individual milk samples according to the two alpine pastures (red square, M_PO = milk from *Poion alpinae*; green dots, M_SE = milk from *Seslerion caeruleae*).

To investigate if the alk profiles could allow the prediction of the milk origin, a PLS‐DA model was proposed. The X‐matrix was composed by the individual milk samples while for Y‐matrix by the 18 retained alks. The details of the model are summarized in Figure 6, while the cross‐validation results of the model are shown in Figure 7 and Table S4. The total explained variance was of 67% per milk pasture and the alks with the VIP > 1 were aconine@17.5, 8‐ethylnorlobelol, 3‐acetyltropine, valerianine@10.1, valerianine@9.0, 8,10‐diethyllobelidiol@12.0, N‐methyllaurotetanine, cinchonanine E, and lycopsamine. Although there is no clear clustering of the milk samples from the two pastures, with a partial overlap in the graph center, the tendency to separate SE samples (left quadrants) from those of PO (right) is still evident. Indeed, 11 samples for PO (46%) and 21 for SE (88%) were correctly reclassified.

## 4 CONCLUSION

A wide and complex range of alks are synthesized by alpine plants and become part of the cow's intake. Despite the difficulty caused by the limited availability of pure commercial standards, this research provided valuable evidence of the varied alk compositions in different pasture herbs. Even though the confirmed alk content in the milk samples was significantly lower than the potential found in plants from mountain pastures, the results were still intriguing.

First, the low transfer rate of toxic alks is reassuring from a health perspective, confirming that milk from cows grazing on mountain pastures poses minimal risk concerning alk toxicity. Second, the comprehensive alk profile examined allowed a notable 67% correct reclassification rate for milk samples from cows grazing in two distinct pastures. This demonstrates the potential for using alk markers as a tool for verifying milk provenance and distinguishing between pasture‐based and industrial farming systems. However, the study's findings should be further investigated due to some inherent limitations concerning the small sample size (number of cows) and the relatively short duration of the study that may have influenced the generalizability of the results.

Moreover, while the differentiation between specific pastures remains challenging at this stage, the presence of alks in milk represents a promising marker for distinguishing pasture‐origin milk from milk produced in intensive plains farming systems. This capability holds significant potential for fraud prevention by authenticating milk origin and ensuring product transparency.

## AUTHOR CONTRIBUTIONS

**Tiziana Nardin**: Writing—original draft; conceptualization; investigation; data curation. **Francesca Martinelli**: Investigation; formal analysis. **Roberto Larcher**: Writing—review and editing; supervision. **Edi Piasentier**: Writing—review and editing; supervision. **Alberto Romanzin**: Investigation.

## CONFLICT OF INTEREST STATEMENT

The authors declare no conflicts of interest.

## Supporting information



Supplementary Information
