# Supplementary material for: Alkaloid profile of Italian alpine milk
Source: J Food Sci. 2025 Feb 4;90(2):e70027. doi: 10.1111/1750-3841.70027 (PMC11792767; doi:10.1111/1750-3841.70027)
Supplement: Supplementary file 1 — Supplementary Information [file JFDS-90-0-s001.docx]

**Table S1**

Individual plant species identified in the experimental Alpine pastures (*Poion alpinae*, PO; *Seslerion caeruleae*, SE), their frequency of occurrence (F, %) in flora assessment replicates and their relative average coverage (AC, %) in pasture.

| **Family** | **Species** | **Common name (Aeschimann et al., 2004)** | **PO** | | **SE** | |
| --- | --- | --- | --- | --- | --- | --- |
|  |  |  | **F** | **AC*** | **F** | **AC** |
| Apiaceae | *Carum carvi* | Caraway | 80 | 0.6 |  |  |
| Apiaceae | *Heracleum sphondylium* | Hogweed | 40 | 0.8 |  |  |
| Apiaceae | *Laserpitium peucedanoides* | (-) |  |  | 56 | 0.8 |
| Asteraceae | *Achillea clavenae* | Austrian Milfoil | 20 | 0.5 | 100 | 1.1 |
| Asteraceae | *Achillea millefolium* | Milfoil | 100 | 0.8 | 22 | 0.4 |
| Asteraceae | *Carlina acaulis* | Stemless Thistle | 20 | 1 | 78 | 0.8 |
| Asteraceae | *Centaurea jacea* | Brown Knapweed |  |  | 56 | 0.7 |
| Asteraceae | *Crepis aurea* | Golden Hawk's-beard | 60 | 1.2 | 40 | 0.4 |
| Asteraceae | *Hieracium pilosum* | Moris' Hawkweed |  |  | 100 | 0.5 |
| Asteraceae | *Leontodon hispidus* | Rough Hawkbit | 40 | 2.3 |  |  |
| Brassicaceae | *Biscutella laevigata* | Biscutella | 80 | 0.4 | 89 | 0.5 |
| Brassicaceae | *Capsella bursa-pastoris* | Shepherd's Purse |  |  |  |  |
| Campanulaceae | *Campanula scheuchzeri* | Scheuchzer's Bluebell |  |  | 44 | 0.4 |
| Cariophyllaceae | *Cerastium arvense* | Field Mouse-Ear | 100 | 1 | 67 | 0.4 |
| Cariophyllaceae | *Dianthus sylvestris* | Wood Pink | 20 | 0.5 | 89 | 0.5 |
| Cariophyllaceae | *Silene alpestris* | Campion |  |  | 56 | 0.4 |
| Cariophyllaceae | *Silene nutans* | Nottingham Catchfly | 60 | 0.5 | 11 | 0.4 |
| Cyperaceae | *Carex atrata* | Black Alpine-sedge |  |  | 11 | 0.4 |
| Cyperaceae | *Carex sempervirens* | Evergreen Sedge | 20 | 0.5 | 89 | 8.7 |
| Dipsacaceae | *Knautia longifolia* | (-) | 20 | 1 | 33 | 0.4 |
| Dipsacaceae | *Scabiosa lucida* | (-) | 40 | 0.8 | 67 | 0.5 |
| Fabaceae | *Anthyllis vulneraria* | Kidney Vetch | 20 | 0.5 | 100 | 1.1 |
| Fabaceae | *Lotus corniculatus* | Common Bird's-foot Trefoil | 40 | 0.5 |  |  |
| Fabaceae | *Medicago lupulina* | Black Medick |  |  |  |  |
| Fabaceae | *Trifolium pratense* | Red Clover | 100 | 3.2 | 33 | 2.9 |
| Fabaceae | *Trifolium repens* | White Clover | 100 | 3.8 | 67 | 1.1 |
| Hypericaceae | *Hypericum maculatum* | Imperforate St John's-Wort | 90 | 2.1 | 78 | 0.4 |
| Juncaceae | *Luzula multiflora* | Heath Wood-rush | 80 | 0.5 | 78 | 0.4 |
| Lamiaceae | *Acinos alpinus* | Alpine Savory | 40 | 0.8 | 78 | 1.6 |
| Lamiaceae | *Lamium album* | White Dead-nettle |  |  |  |  |
| Lamiaceae | *Prunella grandiflora* | Large-flowered Self-heal |  |  | 78 | 8.5 |
| Lamiaceae | *Prunella vulgaris* | Self-heal |  |  |  |  |
| Lamiaceae | *Stachys alopecuros* | Yellow Betony | 40 | 1.8 | 89 | 1.7 |
| Lamiaceae | *Thymus praecox* subsp*.polytrichus* | Wild Thyme | 100 | 2.2 | 100 | 1 |
| Liliaceae | *Veratrum album* | White False-hellebore | 80 | 2.9 | 56 | 0.5 |
| Plantaginaceae | *Plantago atrata* | Mountain Plantain | 100 | 3.8 | 78 | 0.6 |
| Plantaginaceae | *Plantago major* | Greater Plantain |  |  |  |  |
| Plantaginaceae | *Plantago media* | Hoary Plantain | 20 | 0.5 |  |  |
| Poaceae | *Agrostis capillaris* | Common Bent |  |  | 33 | 1.9 |
| Poaceae | *Briza media* | Quaking-grass |  |  | 44 | 3.5 |
| Poaceae | *Dactylis glomerata* | Cock's-foot | 20 | 5 |  |  |
| Poaceae | *Deschampsia caespitosa* | Tufted Hair-grass | 20 | 0.5 | 11 | 0.4 |
| Poaceae | *Festuca calva* | (Fescue) | 20 | 3 | 11 | 0.4 |
| Poaceae | *Festuca rubra* | Red Fescue | 100 | 9.2 | 89 | 9.1 |
| Poaceae | *Phleum rhaeticum* | (Cat's Tail) | 100 | 14.6 | 33 | 1.5 |
| Poaceae | *Poa alpina* | Alpine Meadow-Grass | 100 | 15.4 | 89 | 6.5 |
| Poaceae | *Sesleria caerulea* | Blue Moor-Grass | 20 | 0.5 | 100 | 25.9 |
| Polygonaceae | *Polygonum viviparum* | Alpine Bistort | 40 | 0.5 | 100 | 1.8 |
| Polygonaceae | *Rumex acetosa* | Common Sorrel | 40 | 2.5 | 33 | 0.4 |
| Polygonaceae | *Rumex alpinus* | Monk's Rhubarb |  |  |  |  |
| Ranunculaceae | *Ranunculus acris* | Meadow Buttercup | 100 | 5.8 | 44 | 0.4 |
| Ranunculaceae | *Ranunculus repens* | Creeping Buttercup |  |  |  |  |
| Rosaceae | Alchemilla*gr.vulgaris* | Lady's Mantle | 100 | 4.8 | 56 | 0.5 |
| Rosaceae | *Potentilla crantzii* | Alpine Cinquefoil | 20 | 0.5 | 67 | 2.5 |
| Rosaceae | *Potentilla erecta* | Tormentil |  |  | 22 | 1.1 |
| Rubiaceae | *Cruciata laevipes* | Crosswort |  |  |  |  |
| Rubiaceae | *Galium album* | Upright Hedge-bedstraw | 20 | 0.5 |  |  |
| Rubiaceae | *Galium anisophyllon* | (Bedstraw) | 100 | 0.5 | 89 | 0.4 |
| Scrophulariaceae | *Rhinanthus glacialis* | (Yellow-rattle) | 20 | 0.5 | 100 | 5.4 |
| Scrophulariaceae | *Veronica chamaedrys* | Germander Speedwell | 100 | 1.6 | 22 | 0.4 |
| Urticaceae | *Urtica dioica* | Nettle | 40 | 0.3 |  |  |
| Valerianaceae | *Valeriana* *wallrothii* | Wallroth's Valerian |  |  |  |  |
| *Species without numerical values were sporadic; (-) english name unknown; (name) only genus name known. | | | | | | |

**Table S2**

suspected alk profile of the selected herbage, individual and masses milk from the two experimental pastures and of the mixed concentrate (number of samples containing each individual alk; *Poion alpinae*, PO; *Seslerion caeruleae*, SE). Only alkaloids found in at least one sample are reported.

| Compound | Herbage samples | |  | Individual milk samples | |  | Milk masses samples | |  | Mixed concentrate (N=1) |
| --- | --- | --- | --- | --- | --- | --- | --- | --- | --- | --- |
|  | PO (N=24) | SE (24) |  | PO (N=24) | SE (24) |  | PO (N=6) | SE (6) |  |  |
| Acridinone |  |  |  |  |  |  |  |  |  |  |
| Arborinine | 2 | 1 |  | 0 | 0 |  | 0 | 0 |  | 0 |
| Indole |  |  |  |  |  |  |  |  |  |  |
| Gelsemicine | 3 | 1 |  | 0 | 0 |  | 0 | 0 |  | 0 |
| Gelsemine | 6 | 7 |  | 0 | 0 |  | 0 | 0 |  | 0 |
| Gelsempervine-A/Gelsempervine-C@15.3 | 0 | 3 |  | 0 | 0 |  | 0 | 0 |  | 0 |
| Gelsempervine-A/Gelsempervine-C@15.6 | 0 | 3 |  | 0 | 0 |  | 0 | 0 |  | 0 |
| Harmane@15.9 | 3 | 3 |  | 0 | 0 |  | 0 | 0 |  | 0 |
| Harmol | 5 | 3 |  | 0 | 0 |  | 0 | 0 |  | 0 |
| Isoquinoline |  |  |  |  |  |  |  |  |  |  |
| Caryachine hexoside@13.2 | 4 | 2 |  | 0 | 0 |  | 0 | 0 |  | 0 |
| Caryachine hexoside@16.6 | 6 | 4 |  | 0 | 0 |  | 0 | 0 |  | 0 |
| Caryachine@14.2 | 0 | 1 |  | 0 | 0 |  | 0 | 0 |  | 0 |
| Cheilanthifoline | 3 | 4 |  | 0 | 0 |  | 0 | 0 |  | 0 |
| Cheilanthifoline hexoside@16.5 | 5 | 7 |  | 0 | 0 |  | 0 | 0 |  | 0 |
| Cheilanthifoline hexoside@9.8 | 1 | 2 |  | 0 | 0 |  | 0 | 0 |  | 0 |
| Cheilanthifoline hexoside-hexoside | 0 | 2 |  | 0 | 0 |  | 0 | 0 |  | 0 |
| Escholtzine | 0 | 0 |  | 1 | 0 |  | 0 | 0 |  | 0 |
| Fumaricine | 1 | 0 |  | 0 | 0 |  | 0 | 0 |  | 0 |
| Fumarophycine hexoside | 3 | 2 |  | 0 | 0 |  | 0 | 0 |  | 0 |
| Fumarophycine hexoside-hexoside | 0 | 2 |  | 0 | 0 |  | 0 | 0 |  | 0 |
| o-Methylcaryachine@16.0 | 0 | 1 |  | 0 | 0 |  | 0 | 0 |  | 0 |
| o-Methylcaryachine@18.3 | 0 | 2 |  | 0 | 0 |  | 0 | 0 |  | 0 |
| Parfumidine | 0 | 3 |  | 0 | 0 |  | 0 | 0 |  | 0 |
| Piperidine |  |  |  |  |  |  |  |  |  |  |
| 8,10-Diethyllobelidiol hexoside | 6 | 5 |  | 0 | 0 |  | 0 | 0 |  | 0 |
| 8,10-Diethyllobelidiol hexoside-hexoside@12.8 | 8 | 8 |  | 0 | 0 |  | 0 | 0 |  | 0 |
| 8,10-Diethyllobelidiol hexoside-hexoside@9.9 | 2 | 2 |  | 0 | 0 |  | 0 | 0 |  | 0 |
| 8,10-Diethyllobelidiol pentoside | 0 | 1 |  | 0 | 0 |  | 0 | 0 |  | 0 |
| 8,10-Diethyllobelidiol@12.0 | 3 | 8 |  | 0 | 2 |  | 0 | 0 |  | 0 |
| 8,10-Diethyllobelidiol@13.1 | 0 | 2 |  | 0 | 0 |  | 0 | 0 |  | 0 |
| 8-Ethylnorlobelol | 0 | 3 |  | 0 | 2 |  | 0 | 3 |  | 0 |
| 8-Methyl-10-phenyllobelidiol/Norlelobanidine hexoside | 1 | 2 |  | 0 | 0 |  | 0 | 0 |  | 0 |
| 8-Methyl-10-phenyllobelidiol/Norlelobanidine hexoside-pentoside | 3 | 6 |  | 0 | 0 |  | 0 | 0 |  | 0 |
| 8-Methyl-10-phenyllobelidiol/Norlelobanidine@15.5 | 1 | 4 |  | 4 | 2 |  | 0 | 0 |  | 0 |
| cis/trans Lobelanidine hexoside | 0 | 8 |  | 0 | 0 |  | 0 | 0 |  | 0 |
| cis/trans Lobelanidine hexoside-hexoside@18.9 | 0 | 13 |  | 0 | 0 |  | 0 | 0 |  | 0 |
| cis/trans Lobelanidine hexoside-hexoside@19.7 | 0 | 8 |  | 0 | 0 |  | 0 | 0 |  | 0 |
| cis/trans Lobeline hexoside@17.8 | 4 | 1 |  | 0 | 0 |  | 0 | 0 |  | 0 |
| cis-Lobelanidine/trans-Lobelanidine@20.2 | 0 | 1 |  | 0 | 0 |  | 0 | 0 |  | 0 |
| cis-Lobelanidine/trans-Lobelanidine@20.9 | 1 | 0 |  | 0 | 0 |  | 0 | 0 |  | 0 |
| cis-Lobelanine/trans-Lobelanine@21.2 | 0 | 0 |  | 1 | 0 |  | 0 | 0 |  | 0 |
| Dihydropiperlonguminine | 5 | 2 |  | 0 | 0 |  | 0 | 0 |  | 0 |
| Lelobanidine I/II hexoside@15.6 | 1 | 1 |  | 0 | 0 |  | 0 | 0 |  | 0 |
| Lelobanidine I/II hexoside-hexoside | 4 | 8 |  | 0 | 0 |  | 0 | 0 |  | 0 |
| Lelobanidine I/Lelobanidine II@16.9 | 1 | 2 |  | 0 | 0 |  | 0 | 0 |  | 0 |
| Lelobanidine I/Lelobanidine II@17.4 | 1 | 4 |  | 3 | 1 |  | 0 | 0 |  | 1 |
| Lobinanidine, Isolobinanidine, beta-Lobinanidine@17.6 | 0 | 0 |  | 1 | 1 |  | 0 | 0 |  | 0 |
| Lobinine/Isolobinine@20.0 | 2 | 1 |  | 0 | 0 |  | 0 | 0 |  | 0 |
| Norallosedamine | 6 | 2 |  | 0 | 0 |  | 0 | 0 |  | 0 |
| Norallosedamine pentoside@15.8 | 1 | 7 |  | 0 | 0 |  | 0 | 0 |  | 0 |
| Piperanine | 1 | 0 |  | 0 | 0 |  | 0 | 0 |  | 0 |
| Pyridine |  |  |  |  |  |  |  |  |  |  |
| Ginkgotoxin | 0 | 1 |  | 0 | 0 |  | 0 | 0 |  | 0 |
| Valerianine@10.1 | 0 | 1 |  | 0 | 2 |  | 0 | 2 |  | 0 |
| Pyrrolizidine |  |  |  |  |  |  |  |  |  |  |
| Lycopsamine | 1 | 1 |  | 1 | 0 |  | 1 | 0 |  | 0 |
| Quinoline |  |  |  |  |  |  |  |  |  |  |
| Cinchonanine C@14.8 | 0 | 1 |  | 0 | 0 |  | 0 | 0 |  | 0 |
| Cinchonanine E | 0 | 0 |  | 1 | 0 |  | 0 | 0 |  | 0 |
| Cinchonanine F deoxyhexoside@17.8 | 0 | 1 |  | 0 | 0 |  | 0 | 0 |  | 0 |
| Cinchonanine F hexoside | 1 | 0 |  | 0 | 0 |  | 0 | 0 |  | 0 |
| Cinchonanine F@12.0 | 3 | 7 |  | 0 | 0 |  | 0 | 0 |  | 0 |
| Cinchonanine F@19.1 | 1 | 3 |  | 1 | 0 |  | 0 | 0 |  | 0 |
| Cinchonanine G | 1 | 1 |  | 0 | 0 |  | 0 | 0 |  | 0 |
| Indicain | 0 | 0 |  | 0 | 0 |  | 0 | 0 |  | 1 |
| N-Methyllaurotetanine | 0 | 3 |  | 3 | 1 |  | 0 | 0 |  | 0 |
| Steroidal |  |  |  |  |  |  |  |  |  |  |
| Cevadine@17.2 | 0 | 1 |  | 0 | 0 |  | 0 | 0 |  | 0 |
| Cevadine@18.1 | 6 | 6 |  | 0 | 0 |  | 0 | 0 |  | 0 |
| Terpenoid |  |  |  |  |  |  |  |  |  |  |
| Aconine@17.2 | 5 | 8 |  | 0 | 0 |  | 0 | 0 |  | 0 |
| Aconine@17.5 | 3 | 6 |  | 2 | 0 |  | 0 | 0 |  | 0 |
| Actinidine | 1 | 0 |  | 0 | 1 |  | 0 | 0 |  | 0 |
| Mesaconine@16.3 | 0 | 1 |  | 0 | 0 |  | 0 | 0 |  | 0 |
| Mesaconine@16.6 | 2 | 3 |  | 0 | 0 |  | 0 | 0 |  | 0 |
| Valerianine@9.0 | 0 | 2 |  | 0 | 1 |  | 0 | 0 |  | 0 |
| Valerine | 0 | 2 |  | 0 | 0 |  | 0 | 0 |  | 0 |
| Tropane |  |  |  |  |  |  |  |  |  |  |
| 3-Acetyltropine | 0 | 3 |  | 0 | 2 |  | 0 | 0 |  | 0 |
| Anisodamine | 0 | 1 |  | 0 | 0 |  | 0 | 0 |  | 0 |

**Table S3**

Geographical classification of pastures according to their alkaloid profile using a Partial Least Squares – Discriminant Analysis multiclass model.

|  |  | Binary discrimination of family | | Multiclass |
| --- | --- | --- | --- | --- |
|  |  | PO | SE | overall classification^1^ |
| Confusion matrix (no. of validation samples) | | |  |  |
| true positive (tp_i_) | | 24 | 23 |  |
| false negative (fn_i_) | | 0 | 1 |  |
| true negative (tn_i_) | | 23 | 24 |  |
| false positive (fp_i_) | | 1 | 0 |  |
| Performance evaluation^1^ | | |  |  |
| sensitivity | | 1 | 0.96 | 0.98 |
| specificity | | 0.96 | 1 | 0.98 |
| (average) accuracy | | 0.98 | 0.98 | 0.98 |
| ^1^ Sensitivity_i_ = tp_i_/(tp_i_+fn_i_); Sensitivity_µ_= Σ tp_i_/Σ (tp_i_+fn_i_); | | | |  |
| Specificity_i_ = tn_i_/(fp_i_+tn_i_); Specificity_µ_= Σ tn_i_/Σ (fp_i_+tn_i_); | | | |  |
| Accuracy_i_ = (tp_i_+tn_i_)/(tp_i_+fn_i_+fp_i_+tn_i_); Average accuracy= Σ [(tp_i_+tn_i_)/(tp_i_+fn_i_+fp_i_+tn_i_)] /3xc. | | | | |

**Table S4**

Geographical classification of milk samples according to their alkaloid profile using a Partial Least Squares – Discriminant Analysis multiclass model.

|  |  | Binary discrimination of milk | | Multiclass |
| --- | --- | --- | --- | --- |
|  |  | Milk_PO | Milk_SE | overall classification^1^ |
| Confusion matrix (no. of validation samples) | | |  |  |
| true positive (tp_i_) | | 11 | 21 |  |
| false negative (fn_i_) | | 13 | 3 |  |
| true negative (tn_i_) | | 21 | 13 |  |
| false positive (fp_i_) | | 3 | 11 |  |
| Performance evaluation^1^ | | |  |  |
| sensitivity | | 0.46 | 0.88 | 0.67 |
| specificity | | 0.88 | 0.54 | 0.67 |
| (average) accuracy | | 0.67 | 0.71 | 0.67 |
| ^1^ Sensitivity_i_ = tp_i_/(tp_i_+fn_i_); Sensitivity_µ_= Σ tp_i_/Σ (tp_i_+fn_i_); | | | |  |
| Specificity_i_ = tn_i_/(fp_i_+tn_i_); Specificity_µ_= Σ tn_i_/Σ (fp_i_+tn_i_); | | | |  |
| Accuracy_i_ = (tp_i_+tn_i_)/(tp_i_+fn_i_+fp_i_+tn_i_); Average accuracy= Σ [(tp_i_+tn_i_)/(tp_i_+fn_i_+fp_i_+tn_i_)] /3xc. | | | | |
